# Supplementary figures and images for: Identification of Salt Tolerance Related Candidate Genes in ‘Sea Rice 86’ at the Seedling and Reproductive Stages Using QTL-Seq and BSA-Seq
Source: Genes (Basel). 2023 Feb 10;14(2):458. doi: 10.3390/genes14020458 (PMC9956910; doi:10.3390/genes14020458)

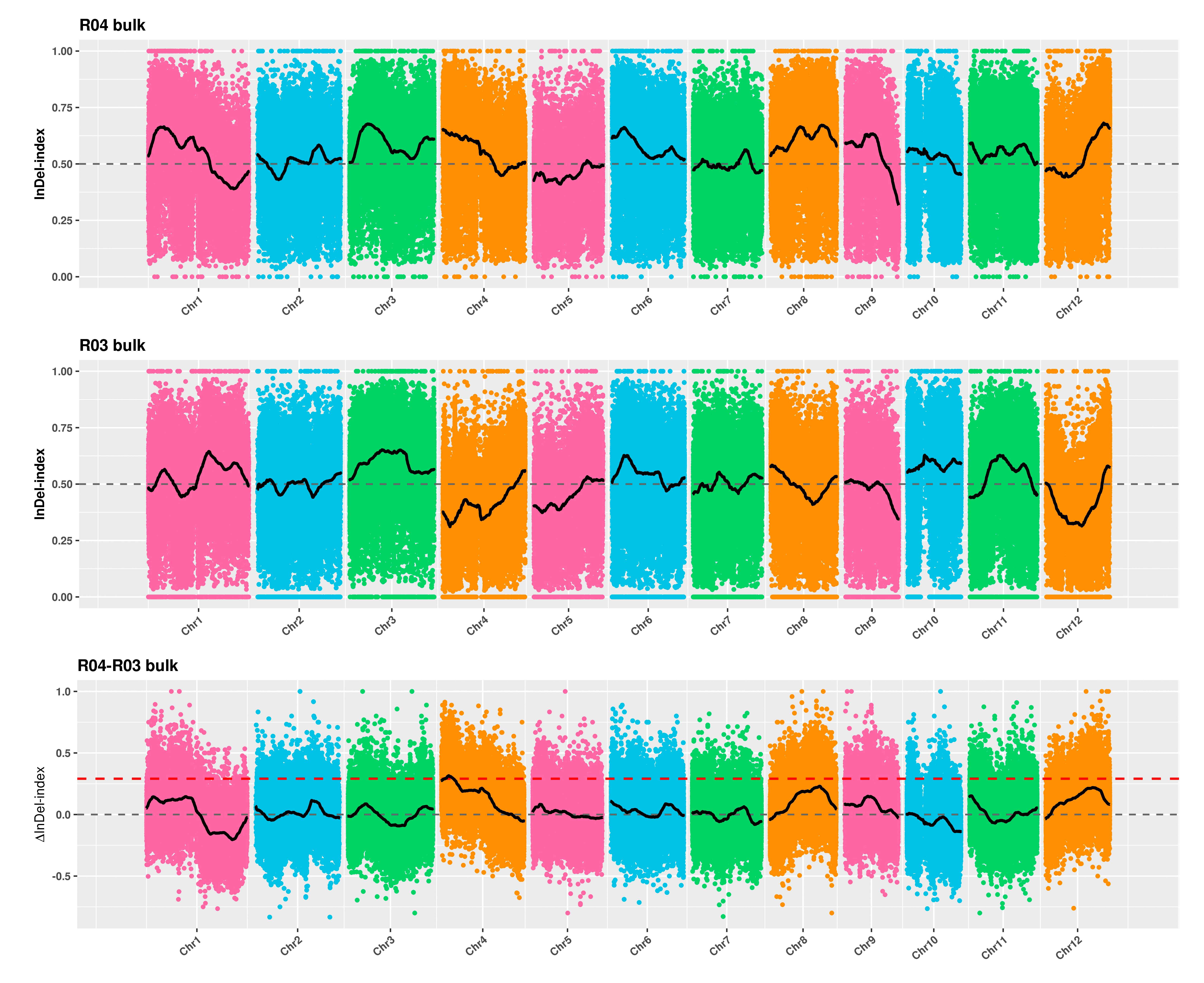

Supplement: Supplementary file 1 [file genes-14-00458-s001.zip › Figure S1.jpg]
